# (Errors in statistical tests)^3^

**DOI:** 10.1186/1742-7622-5-9

**Published:** 2008-07-14

**Authors:** Carl V Phillips, Richard F MacLehose, Jay S Kaufman

**Affiliations:** 1 Department of Public Health Sciences, University of Alberta School of Public Health, 8215 112th St, Suite 215, Edmonton, Alberta, T6G 2L9, Canada; 2 Biostatistics Branch, National Institute of Environmental Health Sciences, National Institutes of Health, PO Box 12233,(MD A3-03), RTP, NC, 27709-2233, USA; 3 Department of Epidemiology, UNC School of Public Health, 2104C McGavran-Greenberg Hall, Pittsboro Street, CB#7435, Chapel Hill, NC, 27599-7435, USA

## Abstract

In 2004, Garcia-Berthou and Alcaraz published "Incongruence between test statistics and P values in medical papers," a critique of statistical errors that received a tremendous amount of attention. One of their observations was that the final reported digit of p-values in articles published in the journal *Nature *departed substantially from the uniform distribution that they suggested should be expected. In 2006, Jeng critiqued that critique, observing that the statistical analysis of those terminal digits had been based on comparing the actual distribution to a uniform *continuous *distribution, when digits obviously are discretely distributed. Jeng corrected the calculation and reported statistics that did not so clearly support the claim of a digit preference. However delightful it may be to read a critique of statistical errors in a critique of statistical errors, we nevertheless found several aspects of the whole exchange to be quite troubling, prompting our own meta-critique of the analysis.

The previous discussion emphasized statistical significance testing. But there are various reasons to expect departure from the uniform distribution in terminal digits of p-values, so that simply rejecting the null hypothesis is not terribly informative. Much more importantly, Jeng found that the original p-value of 0.043 should have been 0.086, and suggested this represented an important difference because it was on the other side of 0.05. Among the most widely reiterated (though often ignored) tenets of modern quantitative research methods is that we should not treat statistical significance as a bright line test of whether we have observed a phenomenon. Moreover, it sends the wrong message about the role of statistics to suggest that a result should be dismissed because of limited statistical precision when it is so easy to gather more data.

In response to these limitations, we gathered more data to improve the statistical precision, and analyzed the actual pattern of the departure from uniformity, not just its test statistics. We found variation in digit frequencies in the additional data and describe the distinctive pattern of these results. Furthermore, we found that the combined data diverge unambiguously from a uniform distribution. The explanation for this divergence seems unlikely to be that suggested by the previous authors: errors in calculations and transcription.

## Background

In 2004, Garcia-Berthou and Alcaraz [GBA] published "Incongruence between test statistics and P values in medical papers [1]." This article reported that last digits of published test statistics and p-values in a sample of consecutive articles from *Nature *deviated from a uniform distribution more than would be expected by chance. The article, which also examined incongruence between reported statistics and p-values, attracted a great deal of attention among journal editors, the popular press, and a large number of readers [2]. In 2006, however, Jeng pointed out that the GBA analysis of last digits was based on a faulty statistical model [3]. The model GBA used tested the distribution of digits against a null hypothesis of a continuous uniform distribution, rather than the 10-bin discrete uniform distribution that is appropriate for the ten possible terminal digits. When reanalyzed in Jeng's critique of the GBA critique, the data that generated the original critique appeared to show rather different results.

Jeng's contribution was one of the more intriguing articles in the health science literature of late, by virtue of being a statistical critique of a statistical critique. It is commendable that he was the first to point out this apparent flaw in an article that had already been accessed at the BioMed Central website over 25,000 times. However, we are concerned by some of the implications of the approach and of the conclusions, both of which reinforce some unfortunate tendencies in the contemporary biomedical literature. In particular, the original GBA critique, as well as Jeng's subsequent critique of their critique, are characterized by excessive attention to statistical significance. Furthermore, both critiques neglect the more fundamental issue that models are either *good enough *or *not good enough*, with the distinction between these judgments being somewhat subjective, rather than *right *versus *wrong *in some absolute sense. Finally, neither article mentions publication bias, which is likely to be an important contributor to digit preference and which has much broader significance for the quality of the health science literature as a whole than do the occasional computational errors that GBA focus on. In response to these concerns, we offer the following critique of a critique of a critique, or in keeping with the title of this analysis, a critique^3^.

## Discussion

One of the best established tenets of modern biomedical statistics [4-6] is that results should not be reported as merely statistically significant or not, as was the common practice decades ago and is still woefully too common in some allied sciences. Although the journal that produced the data used in these critiques, *Nature*, is not primarily a health science journal, the critiques appeared in a journal devoted to research methods in the health sciences, and so it is reasonable to hold those analyses to the standards of modern epidemiology and biostatistics. For the association between two scalars or dichotomous variables (e.g., effect estimates in risk-factor studies), confidence intervals are easy to calculate and report, and are the preferred statistics, as noted by GBA in their article. For some analyses, such as tests of trend and tests of homogeneity, there is no confidence interval to be calculated and a p-value may be a convenient summary [7]. But even in these situations it is clearly deficient to revert to a naïve comparison of p-values to an arbitrary 0.05 criterion as if that were definitive, without any discussion of power or study biases.

Jeng [3] observed that after substituting a chi-square test for the apparently inappropriate Kolmogorov-Smirnov test used in the original critique when examining the distribution of final digits of published test statistics, the tests for departures from the null hypothesis (of a discrete uniform distribution of digits) change from p < 0.0005 in the original critique to p = 0.69. This is obviously quite a dramatic change. But it is more instructive to consider the corresponding re-analysis of final digits in the reported p-values, which generated p = 0.043 in the original analysis and p = 0.086 in Jeng's proposed correction.

Since the subject at hand is interpreting statistics correctly, what sensible interpretation should be made of this? For a p-value of 0.043, we should conclude that it is rather unlikely that we would have seen a deviation this large (or larger) from the null distribution due to sampling variability alone. For a p-value of 0.086 we should conclude pretty much the same thing. Every first-semester epidemiology student learns that there is nothing magical about the p < 0.05 criterion. It is an arbitrary cut-point for a statistic that captures only one of many possible sources of error, and thus it makes no sense to treat it as a bright line. Jeng's statement that, "This changes the results from 'significant' to 'not significant,' and we therefore have insufficient evidence to suggest terminal digit errors in the p values reported in *Nature *articles," reflects an inappropriate deference to the arbitrary demarcation. The two p-values are very similar and their interpretations should not differ very much from each other. Suggesting otherwise reinforces a much more common statistical error in the health sciences – interpreting non-significant results as null results - an error of greater consequence than any discussed by the authors in this series of papers.

Null-hypothesis significance testing may be considered appropriate in situations when we are primarily interested in assessing whether a certain phenomenon exists, regardless of the magnitude [7]. This is rarely the case in health science research, however, where the magnitude of effect matters and hypothesis testing is typically more of a hindrance to good scientific inference [8]. For the analysis in question, it may be reasonable to assume there is a decision to be made about whether there is something "wrong" with the results published in *Nature*. The editors of that journal, for instance, needed to make a decision whether or not to alter their current publication practices to make reporting errors less likely. If the authors truly think a hypothesis test is necessary to make that decision, the first step in performing one should be in identifying the appropriate null hypothesis.

### Is this a reasonable null hypothesis?

Implicit in the decision to perform a null hypothesis significance test is the tentative acceptance of the null hypothesis until such time as evidence from the study would warrant rejection of the null in favor of the alternative hypothesis. The significance testing approach considers the null unlikely only if the observed results (or more extreme results) would not likely arise from sampling variability alone if the null were true. The first critique by GBA attributes the discrepancy they observed (particularly the non-congruence of test statistics and p-values) to transcription errors, typographical errors, and possibly calculation errors – simple goofs that are presumably innocent and random. How such factors could result in an uneven distribution of terminal digits was not addressed and remains unclear. But the null in this scenario might actually be rejected without even having to observe any data at all. In a subsequently published note, Senn observed that even without any improprieties in study conduct, the distribution of terminal digits cannot be expected to be uniform when the null is not true [9]. Furthermore, digit frequencies will additionally be distorted by a deficit of zeros resulting from the common but inappropriate practice of dropping the zero as a final digit when it would have been printed if it were non-zero.

An additional mechanism that likely further distorts observed frequencies of terminal digits is publication bias. Under a simple model for publication bias, those papers with statistically significant results are more likely to get published. For example, if authors report two significant digits for small p-values and the journal is more likely to publish significant results, then where authors are using a 0.05 level of statistical significance we would expect over-representation of low digits (i.e., reported values of 0.01, 0.02, 0.03, 0.04) and under-representation of larger digits. A desire to make results appear statistically significant might also create the opposite effect, with reported values in the range of 0.052 rounded to 0.05. On the other hand, careful attention to the arbitrary 0.05 significance criterion might cause a deficit of 5 s if authors avoid reporting this specific value because they want to be clear about which side of the bright line they are on; instead of reporting 0.05, they expand the reported figure to the next decimal place (e.g., 0.048 or 0.053), while they would not hesitate to print the numbers 0.04 or 0.06.

The expected distribution of terminal digits resulting from all these potential mechanisms acting simultaneously is difficult to predict, but what appears inarguable is that the digit frequencies can reasonably be expected to differ without invoking any calculation errors on the part of authors. Therefore, quarrelling over whether the correct p-value is 0.042 or 0.086 would seem pointless. Even aside from the fact that p = 0.042 and p = 0.086 have virtually identical interpretations in terms of relative likelihoods for the hypotheses being considered [10], it is clear from the above discussion that when the null is almost certainly false, that to attain the coveted p < 0.05 criterion, all one need do is to collect more data, as we do in the next section. In doing so, we might learn something much more informative that we are blinded to by the declaration of "significance". In particular, since we have several stories about the form the digit preference might take, collecting additional data allows us to examine and attempt to interpret the actual digit patterns, rather than blindly conducting a statistical test with a binary result.

### Gathering more data and examining it more closely

We reviewed volumes 413–415 of the online version of *Nature*, and recorded every p-value for which a test statistic was also reported that appeared in all Brief Communications, Review Articles, Articles, and Letters to Nature, for a total of 190 p-values. The original critique by GBA was based on a review of volumes 409–412, which generated 181 p-values. Our data included p-values that appeared in supplementary issues of the journal and excludes all values reported as inequalities. We analyzed this data, as well the new data combined with GBA's original data, using χ^2 ^tests as recommended by Jeng. Analyses were performed in R.

As shown in Table 1, the main result, the χ^2 ^test for whether the terminal digits are consistent with a discrete uniform distribution, yields p < 0.001 for our data and p < 0.001 for our data combined with the original GBA data.

**Table 1:** Comparison of Original GBA Data with New Data and Combined Data

		Terminal Digit of P-Value (Frequency)										
			
Source	n	0	1	2	3	4	5	6	7	8	9	χ^2 ^p-value*
Nature Vols 409–412 (GBA 2004)	181	10	20	25	24	12	16	25	20	16	13	0.086
Nature Vols 413–415	190	14	34	22	27	25	15	17	11	16	9	< 0.001
Combined (Nature Vols 409–415)	371	24	54	47	51	37	31	42	31	32	22	< 0.001
Significant Results Only (p < 0.05)**												
Nature Vols 409–412 (GBA 2004)	85	2	12	15	17	7	7	11	8	5	1	0.001
Nature Vols 413–415	85	1	22	10	16	12	6	6	2	6	2	< 0.001
Combined (Nature Vols 409–415)	170	3	34	25	33	19	13	17	10	11	3	< 0.001
Nonsignificant Results Only (p ≥ 0.05)												
Nature Vols 409–412 (GBA 2004)	96	8	8	10	7	5	9	14	12	11	12	0.646
Nature Vols 413–415	105	13	12	12	11	13	9	11	9	10	7	0.956
Combined (Nature Vols 409–415)	201	21	20	22	18	18	18	25	21	21	19	0.988

* The null hypothesis is that the terminal digits have a discrete uniform distribution

** Due to small sample sizes in some cells, the p-values were calculated through Monte Carlo simulation[16].

In practical terms, conducting the additional data collection and analysis required a relatively modest amount of time and effort. Thus, it was unfortunate that Jeng's critique stopped at the point of finding the existing data to be somewhat ambiguous, and declaring the previous result to be wrong. With a little bit more data, which was there all along for anyone interested, the readily predictable significant deviation from uniformity is confirmed with a very high degree of confidence. Having doubled the sample size, our analysis tends to support the original conclusion, that there is a strong digit preference.

More importantly, neither the GBA critique nor Jeng's critique of GBA's critique seemed particularly interested in the pattern of digit frequencies observed. The reason may have to do with the nature and logic of null hypothesis significance testing, which tends to view sampling variability as the only alternate explanation for a research finding, and thus does not encourage exploration of other potential explanations like publication bias. Looking at the observed pattern, rather than just the test statistic, tends to support some specific interpretations. For the combined data, the departure from the null hypothesis is most readily apparent in a low number of 0 s, as predicted. There is also a shortage of the higher digits and a surplus of digits 1–3 which could be the result of publication bias, and a shortage of 5 s, which could result from the mechanism we proposed.

Since some of the plausible mechanisms through which final digits are favored or disfavored revolve around statistical significance and the absolute magnitude of the statistic, we might learn more about the source of non-uniformity by stratifying. As shown in Table 1, just over half of the reported p-values (54.7%) in both data sets were greater than or equal to 0.05, and as expected these exhibited a distribution that is highly consistent with a uniform distribution (χ^2 ^test for uniform distribution of final digits; p-value = 0.988), while a strong departure from uniformity is found for the p-values of less than 0.05 (χ^2 ^test for uniform distribution of final digits; p-value < 0.001). To some extent, the deficit of large terminal digits among p-values < 0.05 is an obvious artifact of the stratification (when authors report exactly two digits to the right of the decimal, the larger digits are excluded by construction), but the lack of an offsetting surplus of those digits in the other stratum limits this explanation. There seems to be a systematic pattern, not just the sampling variability or random transcription and calculation errors proposed by the previous authors. To some extent, scientists choose to study phenomena that they expect will be demonstrated by the data, a subjective process that creates some bias toward studies having statistically significant results. But given how often a particular study fails to generate the expected result to a strong degree, publication bias on the part of the journal and selective reporting by the authors (i.e., in choosing which test results to display and how many digits to display [11]) seem likely to be major contributors to the observed patterns.

It is important to bear in mind that while Jeng's model may be a substantial improvement over the original continuous-value model, it is still a model with strong assumptions. For example, it assumes that the terminal digits should follow a uniform distribution in the absence of errors, a premise refuted by Senn [9]. Even if Jeng's model were considered reasonable enough to produce an informative analysis, the implication of the dialog (and almost all such dialogs in biostatistics and epidemiology) is that the initial analysis was *right*, until it was proven to be *wrong*, at which point the second analysis was assumed to be *right*. If it were more widely understood that all results were subject to various possible errors, known and unknown, beyond the sampling variability that is reflected in the p-value, we would avoid the absurdity of declaring something to be almost certainly right until the point that we declare it to be completely wrong. Stepping back from these categorical assertions of truth would dramatically improve the conduct, description and interpretation of biomedical research.

### What important sources of error are we neglecting?

Oddities in the last digits of reported p-values may have fairly trivial practical implications, but may be symptomatic of much more important problems of reporting and review that have received limited attention in this discussion so far. Misprinting a chi-square statistic as 1.70 rather than the intended value of 1.07 (an example cited in the original GBA critique) is obviously undesirable, but is really not all that troubling. Such errors tend not to propagate through the rest of the analysis and, assuming they are just random transcription errors, they are non-differential with respect to the true value. Misrepresenting a relative risk estimate as 1.70 rather than an intended value of 1.07 could be much more consequential, however. While a primary study result might be subject to sufficient scrutiny that such a mistake would be unlikely, typically dozens of effect estimates are reported in a published study, any of which might be introduced into a later policy analysis.

Sadly, such errors are unlikely to be detected during a typical peer review. Part of the problem is that peer reviewers seldom have access to the data and analytic model (e.g., software coding), and thus cannot actually check the most important parts of the analysis they are supposed to be vetting, nor would they realistically have time to do so. Even when they can get their hands on the data, the reporting of statistical methods in epidemiology and some other areas of health research is usually inadequate to be able to replicate the analysis. Jeng managed to diagnose exactly what statistic GBA used (to confirm their calculation was based on the null hypothesis of a continuous distribution), but this is only because they published their data (to the credit of these authors and the journal) and were doing a fairly simple analysis. This is a rare circumstance, although published re-analyses do appear occasionally [12].

Furthermore, even with full access to data and models, many errors will escape the scrutiny of whatever reviewers and editors vet a publication. In the present case, for example, the original reviewers missed the opportunity to catch the same error that Jeng did, and all parties involved (including ourselves) missed the point made later by Senn that the distribution on final digits would not be uniform even in the absence of errors [9]. As journal reviewers and editors ourselves, we are constantly worried about out limited capacity to make sure that we do not publish something that warrants a later correction or critique. Thus, we do not intend a critique of individuals, but an observation that the finite capacities of a few busy people will inevitably let important errors escape notice. Clearly, more needs to be done to allow critical readers the chance to continue the peer review after a paper is published [13].

The "soul searching" that the editors of *Nature *reportedly engaged in following the original critique's publication resulted in several changes in practice that might reduce the observed problems, as well as and others that their own audit uncovered [14]. But these primarily took the form of enforcing sensible guidelines about what is reported. For example, sample sizes should be always reported, but this apparently had not always occurred. Regrettably, the new guidelines proposed for *Nature *journals miss the broader points about the utility of hypothesis testing in a mechanical fashion, and how this may be a more important problem than transcription and typographical errors. For example, the new guidelines object to one-tailed tests and insist on corrections for multiple comparisons, strategies which merely require the choice of one particular option among several flawed approaches. While newly instituted recommendations might allow peer reviewers or subsequent critical readers to catch a few more of the minor errors, there are many more important errors that remain just as encased in black boxes as ever.

## Conclusion

The problems identified in this chain of critiques tend to undermine the credibility of published biomedical research, perhaps with good reason. The best solution to the problem is to expose more of the process to scrutiny by the community of scientists, allowing peer review to accomplish what it is supposed to. With more responsible publication that includes disclosure of all relevant information in an operationalizable form, including data and the details of what models were used and why, mistakes will more likely be identified eventually by readers, and thus probably avoided to a much greater extent in the first place. Also needed, however, is more willingness on the part of journals to publish re-analyses of previously published results rather than a stream of disjoint monologues. Jeng's paper was so delightful to read in part because it is rare to see critical scientific interchange in the health science literature.

In their conclusions, GBA argue that, " [s]purious precision adds no value to a paper and even detracts from its readability and credibility". This same point was previously argued by one of us in the same journal a year earlier, based on a problem GBA so ironically illustrated: uncertainty about errors in models means that the reported precision is seldom justified [15]. Jeng took this further, writing, "While their paper still points to the need for greater scrutiny of statistics, that scrutiny would be better directed at the assumptions used in the statistical tests, rather than at the precise p-values obtained." We agree with these authors, and for round three, we venture to take this critique a step further, suggesting that greater scrutiny by empowered readers of all aspects of our analyses is the only practical way to deal with statistical errors, as well as a host of other kinds of errors and other limitations of published results.

## Abbreviations

GBA: Garcia-berthou and alcaraz (authors of "Incongruence between test statistics and P values in medical papers", *BMC Medical Research Methodology*, 2004).

## Competing interests

The authors declare that they have no competing interests.

## Authors' contributions

The authors jointly conceived of the paper and jointly developed the analysis through a series of discussions. CVP drafted the initial version of the text all authors participated in several rewrites. RFM conducted the statistical analysis.
